# Asymmetric Cross Metasurfaces with Multiple Resonances Governed by Bound States in the Continuum

**DOI:** 10.3390/ma16062227

**Published:** 2023-03-10

**Authors:** Hongjie Fan, Jing Li, Yuhang Sun, Xueyu Wang, Tiesheng Wu, Yumin Liu

**Affiliations:** 1 State Key Laboratory of Information Photonics and Optical Communications, Beijing University of Posts and Telecommunications, Beijing 100876, China; 2 College of Information and Communication Engineering, Guilin University of Electronic Technology, Guilin 541004, China

**Keywords:** all-dielectric metasurface, bound state in the continuum, optical switching, sensing

## Abstract

The bound state in the continuum (BIC) has paved a new way to achieve excellent localization of the resonant mode coexisting with a continuous spectrum in the metasurface. Here, we propose an all-dielectric metasurface consisting of periodic pairs of asymmetric crosses that supports multiple Fano resonances. Due to the sufficient degrees of freedom in the unit cell, we displaced the vertical bars horizontally to introduce in-plane perturbation, doubling the unit cell structure. Dimerization directly resulted in the folding of the Brillouin zone in k space and transformed the BIC modes into quasi-BIC resonances. Then, simultaneous in-plane symmetry breaking was introduced in both the x and y directions to excite two more resonances. The physical mechanisms of these BIC modes were investigated by multipole decomposition of the scattering cross section and electromagnetic near-field analysis, confirming that they are governed by toroidal dipole (TD) modes and magnetic dipole (MD) modes. We also investigated the flexible tunability and evaluated the sensing performance of our proposed metasurface. Our work is promising for different applications requiring stable and tunable resonances, such as optical switching and biomolecule sensing.

## 1. Introduction

Metasurfaces have attracted considerable attention due to their ability to control light propagation and light localization [1,2,3]. Using design methods for structural parameters of metacells, metasurfaces can be developed for many applications, such as beam splitters [4], holograms [5], ultra-narrow-band absorbers [6,7], filters, sensors [8,9], etc. A key concept is the generation of the high quality factor (Q factor). Metasurfaces provide a versatile platform to achieve high Q-factor resonances via suitable material selection and unique designs due to the advantages of multicell interactions in periodic arrays [10,11,12]. Recently, the bound state in the continuum (BIC) has provided a novel method to achieve high Q-factor resonances with exotic optical properties in metasurfaces [13,14,15,16,17,18,19,20].

In contrast to the resonance modes caused by couplings between light waves and the radiation continuum, the BIC remains perfectly confined in the continuum and coexists with other radiative waves without any radiations [16], which defies conventional wisdom. Derived from quantum mechanics, the BIC is widely observed in the results of interactions between different material systems and various types of waves, such as acoustic [21] and optical [22] waves. In optics, an ideal BIC is decoupled from other radiative modes so that it is optically unobservable due to its zero linewidth and infinite Q factor [15]. In different metasurface systems, BICs can be divided into two typical patterns: symmetry-protected BICs and accidental BICs [3]. The latter can realize radiation suppression by tuning the structural parameters when the number of radiative channels is small. This suppression can be regarded as destructive interference among radiative modes [23,24]. The former forbids couplings between resonances and radiative channels when the symmetry is preserved. When symmetry breaking is introduced in the system, an ideal BIC can be converted into a quasi-BIC, which can leak into the external continuum so that it can be observed in the spectrum in the form of sharp Fano resonance with narrow linewidth and a finite high Q factor [11,15,25,26,27]. Furthermore, the Q factors of quasi-BICs are decided by the magnitude of the perturbation. Previous works have provided structural asymmetry or electromagnetic field asymmetry to realize high Q-factor resonances by oblique incidence [28] or by introducing defects in metacells of metasurfaces, such as dielectric nanodisks with off-center holes [29], blocks with defects [30,31], and split-ring resonators [32]. However, the limited degrees of freedom that can be adjusted in simple cells only excite one or two resonances in a single band, restricting their applications in multiband devices. Out-of-plane symmetry breaking, as a new degree of freedom, has also been investigated in the G-Hertz band, but this design can be a challenge for fabrication at the nanoscale [33]. Many multimer-based metasurfaces have been proposed to better control the resonances in the spectra, such as asymmetric nanodisk tetramers [7] or unequal bars [22,34] and asymmetry nanohole arrays [35]. However, these designs with different defects or holes induce changes in the total refractive indices of the structure, which further cause distinct shifts in spectral resonant wavelengths to varying degrees. In practical applications, a controllable Q factor is important, but a relatively stable resonant wavelength is also in demand. The tunability of the designed metasurface has also been considered in some works [36], many geometric parameters, including the periodicity, must be changed to achieve tuning for the resonant wavelength, which is not conducive to the practical needs in limited space. As a result, as a practical consideration, it is also essential to propose easily tunable metasurfaces supporting multiple resonances.

In this paper, we propose an all-dielectric metasurface supporting multiband sharp Fano resonances. The unit cell is composed of a pair of dielectric crosses, which provides sufficient degrees of freedom for the study of multiple Fano resonances. First, we introduce in-plane perturbation by displacing the vertical bars along the *x*-axis to achieve the conversion of the BIC to a quasi-BIC. After the introduction of perturbation, the period of original structure is doubled, the Brillouin zone is folded to half of its original extent, and the number of eigenstates at point Γ is doubled, which makes it possible to excite more Fano resonances compared to a single unit cell. Since this method does not perturb the total refractive index of the system, there is almost no fluctuation in the resonance wavelengths for either resonance during the manipulation of Q factors by changing the magnitude of the perturbation. We also study the flexible tuning of the resonant modes under the influence of different geometrical conditions and investigate the performance as a function of optical switching. In our structure, we do not need to change many geometric parameters, including the period for tuning, which greatly increases the convenience of tuning and can ensure that tuning can still be completed in the case of limited geometric space. Then, the asymmetric perturbations in both the x and y directions are introduced to excite more high Q resonances. In addition, considering the difficulty of experimental fabrication, we calculate the Q factors in the case of large asymmetric parameters, demonstrating that the metasurface can still maintain high Q factors. We evaluate the variations of Q factors of these quasi-BIC modes for different incident polarization angles. Finally, the effects of different ambient refractive indices on these four resonant modes are also investigated. Thus, the present work may provide a new reference for high-Q devices requiring stable resonance properties and multiple degrees of freedom and help to develop relevant applications, such as biochemical sensing, optical switches, lasers, etc.

## 2. Materials and Methods

**Figure 1:**
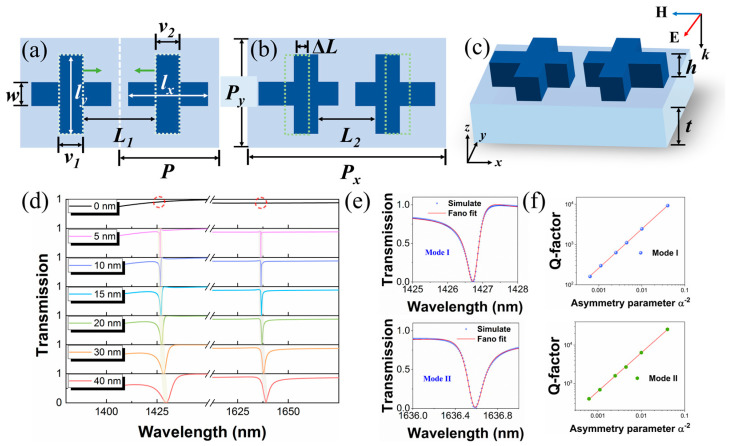
Schematic of the proposed metasurface. (**a**) Original structure of the unit cell. The diagram shows two adjacent unit cells. *P* = 600 nm, *L*_1_ = 450 nm, *w* = *v*_1_ = *v*_2_ = 150 nm, *l_x_* = 500 nm, and *l_y_* = 600 nm. (**b**) The dimer metacell of the metasurface after introducing perturbation. *L*_2_ = 430 nm, *P_x_* = 2*P* = 1200 nm, and *P_y_* = 800 nm. (**c**) *h* = 300 nm, and *t* = 1000 nm. The incident light is a linearly y-polarized plane wave. (**d**) Transmission spectra with varying Δ*L* values. The symbols at the top of the spectra mark the positions of BICs. (**e**) Fano fit curves for the simulation results of modes I and II when Δ*L* is 10 nm. (**f**) Log–log plots of Q factors with respect to the growing asymmetry parameter for modes I and II.

As shown in Figure 1a, a cross is arranged in the center of the original unit cell with a periodicity of *P* in the x direction. The lengths of the orthogonal bars are *l_x_* and *l_y_*, and they share the same width (*w*). The distance of the vertical bars for the adjacent crosses is *L*_1_. Then, these two vertical bars of the adjacent crosses are moved towards the center simultaneously to introduce in-plane perturbation, as shown in Figure 1b. The periodicity in the x direction changes to *P_x_* = 2*P*. Other geometrical parameters remain unchanged. The shaped cross with a height of *h* is on the silica substrate with a thickness of *t*. In the simulation, silicon is set as the building material for the upper cross structure, and silica is chosen as the substrate material due to the negligible optical loss in the common infrared detection waveband. The optical constants refer to Palik’s handbook [37]. The geometric parameters are all shown in Figure 1. The numerical simulations are performed according to the finite-element method (FEM) and finite-difference time-domain (FDTD) method. The periodic boundary conditions are applied in the x and y directions, and perfectly matched layer (PML) boundary conditions are applied in the z direction on the ends of the physical domain. The y-polarized plane wave propagates along the z direction normally incident on the proposed metasurface. The subsequent numerical simulations involving FEM methods described in this paper are implemented using COMSOL Multiphysics™ commercial software (version 5.5).

## 3. Results and Discussions

BIC protected by symmetry has an infinite Q factor due to the couplings between the resonance mode and external radiation channels are forbidden. However, when these two vertical arms are moved towards the center, in-plane perturbation is introduced in the system, which results in leaky modes in free space. To quantify the magnitude of the perturbation, we use Δ*L* = (*L*_1_ − *L*_2_)/2 to reveal it and set dimensionless parameter α = Δ*L*/1 (nm) as the asymmetry parameter. The transmission spectra are calculated in Figure 1d with varying Δ*L* values from 0 nm to 40 nm. Clearly, there are two resonance dips, labeled as mode I and II, that can be observed in the spectra. As Δ*L* decreases, the resonant linewidths get narrower, and the resonance dips simultaneously become sharper. When the asymmetry parameter approaches zero, two resonances disappear completely, which means that the couplings between resonant modes and radiative channels are forbidden. The positive correlation between coupling strength and perturbation indicates the existence of symmetry-protected BICs. As perturbation is introduced, these two BICs are converted into quasi-BICs with narrow resonant linewidths, and two sharp Fano resonances appear. Furthermore, the resonance wavelengths are relatively stable compared with other metasurfaces that include the change of total refractive index caused by different etched holes [26,35] or nanoantennae [38] in systems.

Fano resonance arises from the constructive and destructive interference between a narrow discrete resonance and a spectrally overlapping broad continuum. These conditions can be satisfied when the BIC mode couples to free space [15,39]. Therefore, we plot the spectra when Δ*L* = 10 nm and fit the transmission curves for mode I and mode II using the classical Fano formula, as shown in Figure 1e [40]:

(1) $$T\left(\omega \right)=T_{0}+A_{0}\frac{{\left[q+2\left(\omega -\omega_{0}\right)/\tau \right]}^{2}}{1+{\left[2\left(\omega -\omega_{0}\right)/\tau \right]}^{2}},$$

where *T*_0_ is the transmission offset, *A*_0_ is the coupling constant, *q* is the asymmetry factor, *ω* is the resonant wavelength, and *τ* is the resonant linewidth. The Q factors of resonant modes are calculated by Q = *ω*_0_/*τ*. The calculated Q factors shown in Figure 1e are 2445.5 and 6325.7 for modes I and II, respectively. In addition, in order to reveal the deep relationship between Q factor and the asymmetry parameter, the dependence of Q factors on the asymmetry parameter (*α*) for modes I and II is shown in Figure 1f. One can observe that the radiative Q factor and the asymmetry parameter satisfy the following quantitative relationship:

(2) $$Q\propto \alpha^{-2}.$$

This inverse quadratic relationship further indicates that mode I and mode II are governed by symmetry-protected BICs [15,41]. As shown in Figure 1d, in the absence of perturbation, no resonance peak is observed in the spectrum. When the perturbation is introduced, the non-radiating BIC couples to the free space and is converted into a quasi-BIC. As the asymmetry parameter increases, the Q factor decreases, as shown in Figure 1e, which can be distinguished from the non-BIC state. As Δ*L* is reduced, the Q factors tend to be infinite and can be manipulated by reducing the asymmetry parameter. Previous studies have provided many good references for achieving high Q-factor resonances [42,43]. However, the Q factors in 1D or 2D structures are reduced to varying degrees in practical applications, and the very precise control of the position and size of the nanostructure has high requirements for the fabrication process. Thus, considering the actual technological limitations, we only consider large asymmetric parameters to calculate Q factors. In practical applications, a high spectral contrast ratio is also essential. The spectral contrast ratio (SCR) is defined as [(T*_max_* − T*_min_*)/(T*_max_* + T*_min_*)], where the T*_max_* and T*_min_* are the maximum and minimum at resonances of transmission curves, respectively. In our proposed metasurface, mode I and mode II both show good SCRs, as shown in Figure 1d. When Δ*L* is 10 nm, their SCRs reach up to 99.97% and 99.50%, respectively.

**Figure 2:**
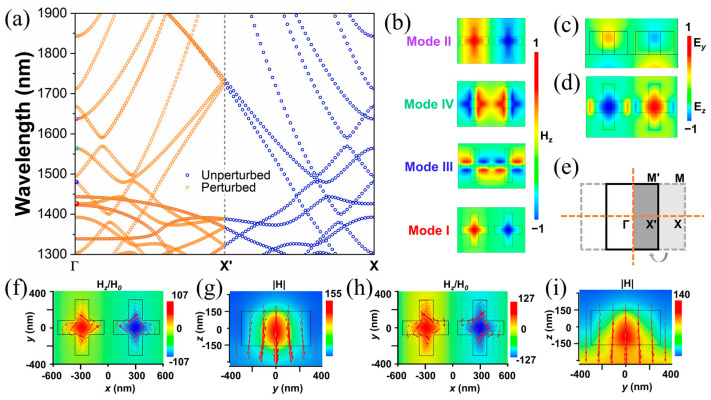
(**a**) Numerically simulated band diagram for unperturbed and perturbed designs. Four target wavelengths are highlighted in color point Γ. The unperturbed geometrical configurations are the same as those in Figure 1a. The magnitude of perturbation is Δ*L* = 1 nm. (**b**) Out-of-plane magnetic field components of the perturbed structure for four target modes. (**c**) The y-component of the electric field for mode IV. (**d**) Out-of-plane electric field component for mode III. (**e**) First Brillouin zone of the unperturbed and perturbed lattices. (**f**–**i**) The electromagnetic field distributions when Δ*L* is 10 nm, corresponding to Figure 1e. The normalized z component of magnetic field distributions with displacement current density at a resonant wavelength for mode I (**f**) and mode II (**h**) in the x–y plane. The normalized magnetic distribution in the y–z plane at x = 300 nm for mode I (**g**) and mode II (**i**).

The band diagram calculated by eigenfrequency analysis is shown in Figure 2a, revealing the key role of the perturbations we introduced in Figure 1a. The band structure is carried out using an FEM eigenfrequency solver. In the simulation, the boundary conditions with Floquet wave numbers *k_x_* = *k*_1_ × 2π/*a*_1_ and *k_y_* = 0 (*k*_1_ ∈ [0, 0.5]) are applied in the unit cell. The lattice constant is *a*_1_ = *P_x_*, where *P_x_* is the period in the x direction of the unperturbed and perturbed structure, as shown in Figure 1a,b. In order to better observe the change in band structure, Δ*L* = 1 nm is taken in the perturbed structure, and other parameters are the same as those in Figure 1. The first Brillouin zone (FBZ) for unperturbed and perturbed designs are shown in Figure 2e, which are enclosed by gray dashed lines and a black solid line, respectively. The high symmetry points relative to the unperturbed and perturbed designs are also included. As these two vertical bars move towards the center simultaneously, the period in the real space is doubled in the x direction, and the extent of the FBZ in the momentum space is compressed accordingly to half of its original size. According to Bloch’s theorem, the eigenstates belonging to the unperturbed FBZ outside the new FBZ are equivalent to the eigenstates in the new FBZ. After the introduction of perturbation, the outer half of the original FBZ (light gray area) is folded into the dark gray area, and the original X point is folded to point Γ, while new point X (X′ point) is at the midpoint of original point Γ and point X. Consequently, the eigenstates at point X are all folded at point Γ, owing to the perturbation. In the perturbed design, the target eigenstates marked by colored points at point Γ in Figure 2a are labeled as modes I, II, III, and IV. The corresponding eigenmode profiles are depicted in Figure 2b,d.

The introduction of perturbation brings the eigenstates at point X to point Γ, which can overlap and couple with the electric field of linearly polarized light at normal incidence. As the asymmetric parameter increases, the coupling increases, and the line width increases accordingly, resulting in the changes shown in Figure 1d. After the introduction of perturbation, the high symmetry point originally located at the center of the single cross is folded to the center of the gap between two crosses as the FBZ is folded. As depicted in Figure 1b, the perturbed 2D periodic unit cell belongs to the point C_2*v*_ group, which is invariant under symmetry operations {E, C_2_, σ_x_, σ_y_}. The mode profiles are irreducible representations of the point group according to the group theory. The C_2*v*_ point group has four non-degenerate presentations: A_1_, A_2_, B_1_, and B_2_. The character table of the point group is shown in Table 1, which can be used to identify the corresponding irreducible presentations. The number 1 indicates a symmetry profile, and -1 represents an antisymmetric profile after operations. We further analyze symmetry of four mode profiles in Figure 2b. Modes I and II show the H_z_ component of the symmetry-protected BICs. The symmetry operations {E, C_2_, σ_x_, σ_y_} corresponding to the character table are {1, −1, 1, −1}. Therefore, modes I and II belong to the B_1_ irreducible representation for the point C_2*v*_ group. Similarly, the symmetry operations {E, C_2_, σ_x_, σ_y_} for modes III and IV are represented by the characters {1, −1, −1, 1} and {1, 1, 1, 1}, which can be recognized as B_2_ and A_1_ irreducible representations, respectively. By investigating the irreducible representation of the four modes, it is easy to find that modes III and IV are decoupled from the radiation continuum due to the mismatch with the incident y-polarized light mode. The B_1_ modes (modes I and II) at point Γ match the magnetic symmetry of the normally incident y-polarized light so that the coupling with the external radiation occurs under the normal y-polarized incidence, and resonances on the spectra can be observed due to perturbations, as shown in Figure 1d.

The electromagnetic near-field distributions at resonant wavelength under the incident condition of normal y-polarized light are plotted in Figure 2f–i. It can be seen that the distributions of the mode profiles remain consistent with the eigenmodes in Figure 2b. The cones in Figure 2f,h represent the displacement current density, and those in Figure 2g,i represent the magnetic field vectors. In Figure 2f,h, we plot the magnetic field patterns of the z component in the x–y plane at resonant wavelengths. Two sets of ring currents symmetrically mirror and circulate around both sides of the centerline, which is the typical distribution for the TD mode. The directions of both TD modes are mostly along the y direction due to the special y-polarized incident light. Figure 2g,i depict the magnetic field patterns in the y–z plane at the center of the right cross in Figure 2f,h, respectively. The magnetic field distributions in the single cross follow the right-hand screw rule according to the displacement current distributions.

In order to further investigate the underlying physical mechanism for these two modes, electromagnetic multipole decomposition method is performed for the dielectric crosses in a Cartesian coordinate system [44,45]. The multipole moments are determined by the light-induced polarization or displacement current (**j**(***r***)), which can be expressed as:

(3) $$j\left(r\right)=-j\omega \left(\varepsilon_{n}-\varepsilon_{b}\right)E\left(r\right),$$

where *ω* is the angular frequency; *ε_n_* and *ε_b_* are the dielectric permittivities of the crosses and surrounding background medium, respectively; ***E****(**r**)* is the total electric field in the dielectric crosses; and ***r*** is the position vector defined as ***r*** = (*x*, *y*, *z*). The dominant multipole moments, including electric dipole (ED), magnetic dipole (MD), toroidal dipole (TD), electric quadrupole (EQ), and magnetic quadrupole (MQ) moments, under the long-wavelength approximation condition are expressed as:

(4) $$P=-\frac{1}{i\omega }\int j\;d^{3}r,$$

(5) $$M=\frac{1}{2}\int \left(r\times j\right)\;d^{3}r,$$

(6) $$T=\frac{1}{10c}\int \left[\left(r\cdot j\right)r-2r^{2}j\right]\;d^{3}r,$$

(7) $$Q_{\alpha \beta }=-\frac{1}{i\omega }\left[\begin{array}{l} \int \left\{3\left(r_{\beta }j_{\alpha }+r_{\alpha }j_{\beta }\right)-2\left(r\cdot j\right)\delta_{\alpha \beta }\right\}d^{3}r\\ +\frac{k^{2}}{14}\int \left\{4r_{\alpha }r_{\beta }\left(r\cdot j\right)-5r^{2}(r_{\alpha }j_{\beta }+r_{\beta }j_{\alpha })+2\left(r\cdot j\right)\delta_{\alpha \beta }\right\}d^{3}r \end{array}\right],$$

(8) $$M_{\alpha \beta }=\int \left[{\left(r\times j\right)}_{\alpha }r_{\beta }+{\left(r\times j\right)}_{\beta }r_{\alpha }\right]d^{3}r,$$

where *k* is the wave number. The total scattering cross section is determined by:

(9) $$\sigma_{sca}\approx \frac{k^{4}}{6\pi \varepsilon_{0}^{2}\left|E_{0}\right|}\left[\sum \left({\left|P+ikT\right|}^{2}+{\left|\frac{M}{c}\right|}^{2}\right)+\frac{1}{120}\sum \left({\left|Q_{\alpha \beta }\right|}^{2}+{\left|\frac{k}{c}M_{\alpha \beta }\right|}^{2}\right)\right].$$

**Figure 3:**
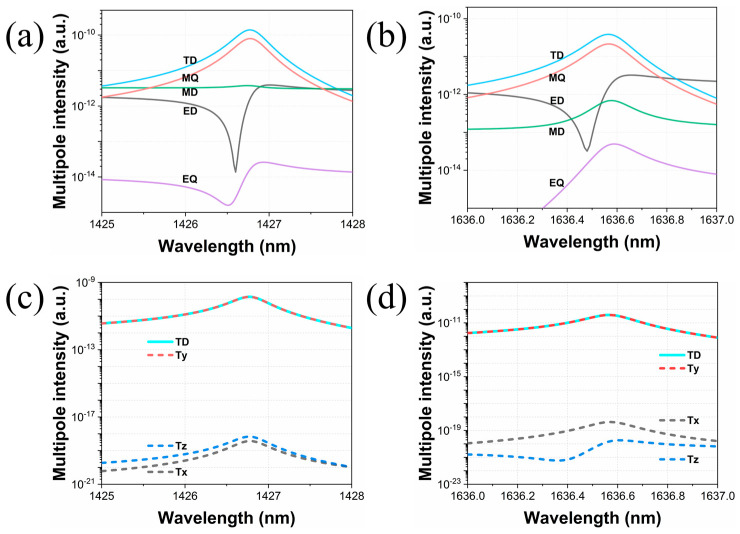
Multipole decomposition around resonant mode I (**a**) and mode II (**b**). The x, y, and z components of TD moments are shown around mode I (**c**) and mode II (**d**).

Where **P**, **M**, **T**, *Q_αβ_*, and *M_αβ_* represent the ED, MD, TD, EQ, and MQ moments, respectively. With the same physical configuration as in Figure 1, we apply the above multipole decomposition method to the dielectric crosses in a unit cell when Δ*L* = 10 nm. The multipoles of the structure are located at the origin of the Cartesian coordinate system and coincide with the center of mass of the structure. In Figure 3, we plot the scattering cross section of multipole moments determined by the induced currents. Although the toroidal dipole moment mathematically appears as expanded terms in series expansions, it has to be taken into account so that the size of our proposed structure is comparable to the incident wavelength. For modes I and II shown in Figure 3a,b, one can observe that TD and MQ moments are dominant components, but TD moments contribute to both resonances. We also decompose the TD moment in three directions in Cartesian system to recognize that TD modes are mainly excited in the y direction according to the almost negligible components in the x and z directions. These results are in agreement with the patterns obtained in the electromagnetic near-field analysis shown in Figure 2.

**Figure 4:**
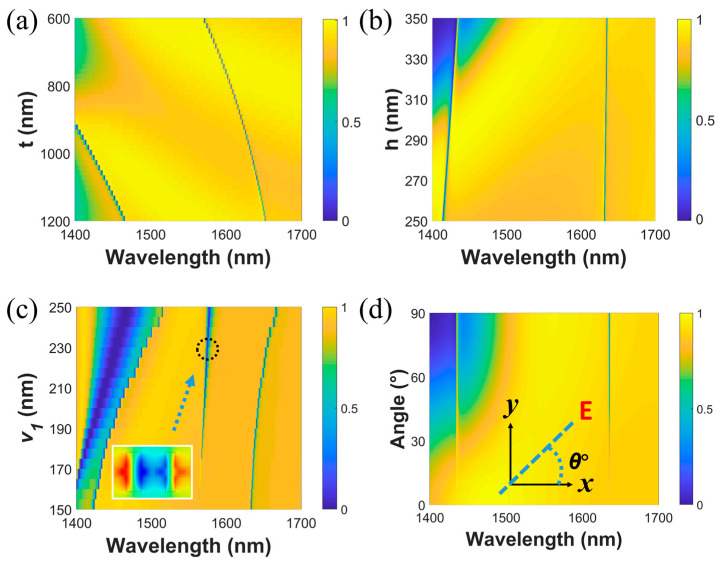
(**a**) Transmission spectra with varying substrate thickness (t). (**b**) Transmission spectra with varying dielectric height (h). (**c**) The calculated transmission spectra when *v*_1_ changes from 150 nm to 250 nm. The inset is the out-of-plane magnetic field component for the new resonance when *v*_1_ is 230 nm. (**d**) Transmission spectra with varying incident polarization angles from 0° to 90°.

To further demonstrate the flexibility and robustness of our proposed metasurface, the transmission responses with different conditions are considered. The analyses are based on the choice of Δ*L* = 10 nm. Figure 4a shows the transmission spectra when the structures are on different substrates with thicknesses varying from 600 nm to 1200 nm. One can observe that modes I and II both redshift due to the increasing total refractive indices of the whole systems. Additionally, the transmission spectra with varying heights of the silicon crosses from 250 nm to 350 nm are plotted in Figure 4b. Obviously, mode I redshifts slightly, while mode II is almost unchanged. This good robustness is very conducive to the actual fabrication process and compatible for integration under different physical constraints. More precise tuning for both modes is also taken into account when the thickness is determined. Figure 4c shows the transmission spectra as the vertical bar of the left cross becomes wider from *v*_1_ = 150 nm to *v*_1_ = 250 nm. Similar to Figure 4a, modes I and II show the same redshift trend due to the increasing effective refractive indices. However, a new stable, quasi-BIC resonance appears in the spectrum due to the symmetry breaking on both sides of the *y*-axis. Figure 4c depicts the out-of-plane magnetic field component for this resonance when *v*_1_ is 230 nm. It can be seen that the field distribution and resonant wavelength are consistent with those of mode IV in Figure 2a,b. Additionally, the linewidth of the resonance keeps increasing with the increase in the width difference between the two arms (*v*_1_–*v*_2_), so the resonance arises from mode IV. The quasi-BIC can be converted into BIC when *v*_1_ and *v*_2_ keep the same width and change at the same time (This resonance mode will be discussed in detail below). In practice, the geometric adjustments of single and double arms can be selected depending on the magnitude of tuning. It is worth mentioning that through our unique design, we find that our proposed metasurface does not need to change many geometric parameters, including the periodicity, during tuning of the resonant wavelength, but only needs to change the bar widths within a unit cell. This design is desirable for cases in which external physical space is limited and high precision tuning is required. The above tunable features based on the substrate and the cross bars make it possible to be applied to different wavelength applications according to actual requirements.

**Figure 5:**
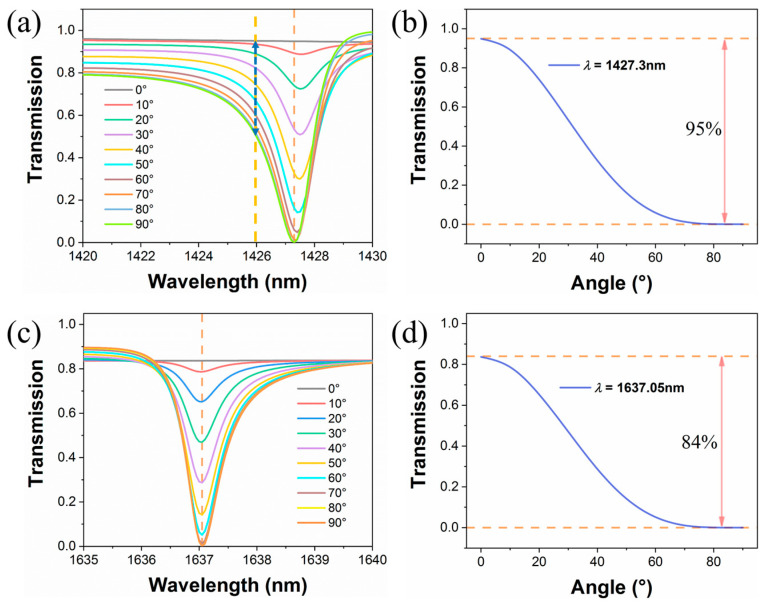
Transmission spectra for modes I (**a**) and II (**c**) with different incident polarization angles. The transmission intensity at wavelengths of 1427.3 nm (**b**) and 1637.05 nm (**d**) with continuously varying polarization angles.

Then, the incident polarization angles are adjusted, and other conditions are unchanged. Herein, the incident polarization angle (*θ*°) is defined as the angle between the *x*-axis and the polarization direction of the incident electric field, as shown in the inset of Figure 4d. Since the symmetry of the mode profiles of modes I and II cannot match with the symmetry of x-polarized normally incident light, these two resonant modes cannot be observed in the spectra under x-polarized normal incidence. This characteristic can be used for the study of optical switching or amplitude modulation. As the polarization angles changes from 0° to 90°, the shifts for the positions of these two resonances in the transmission spectra are almost invisible, which is significant for practical high Q-factor optical detection. As shown in Figure 5, linear changes in polarization angles can lead to corresponding changes in transmission responses at a particular wavelength. For mode I, one can observe that if a plane wave is incident on the proposed metasurface, the transmission intensity of the plane wave exhibits a strong dependence on the polarization angle. Figure 5b presents the dependence between transmission intensity and the polarization angle at a wavelength of 1427.3 nm, and a rotation of the polarization angle from 0° to 90° can cause the transmission intensity to be modulated from 95% to zero. Similarly, for mode II, the dependence relationship between transmission and polarization angle is shown in Figure 5c,d. Supposing that the linearly polarized plane wave is incident to the metasurface, the optical switching effect can be achieved by changing the incident polarization angle at specific wavelengths. In addition, considering that the optical signal is always modulated to a carrier with a narrow bandwidth wavelength in practical optical communication, the high Q-factor and narrow linewidth make it possible to modulate the transmission to different extents in a narrow band, for example, from 50% to 95% at a wavelength of 1426 nm.

**Figure 6:**
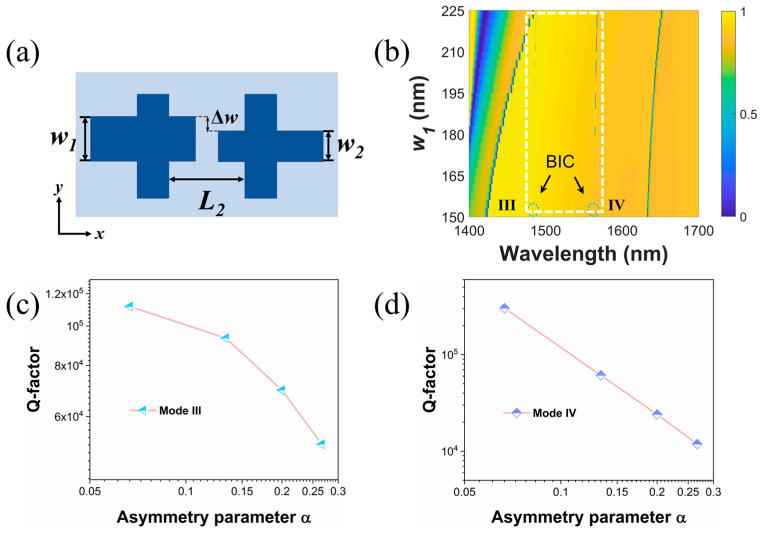
(**a**) Schematic view of the unit cell in the presence of a new perturbation. (**b**) Transmission spectra with increasing *w*_1_. The positions of BICs in the spectra are marked by dashed circles. Log–log plots of Q factors with respect to the varying asymmetry parameter for mode III (**c**) and mode IV (**d**).

In order to observe multiple Fano resonances under the same y-polarized conditions in the dimer metasurface, we try to excite modes III and IV by means of symmetry breaking. As shown in Figure 6a, keeping other conditions in Figure 1b unchanged, when *w*_1_ ≠ *w*_2_, the proposed metasurface is asymmetric with respect to the x and y axes. The transmission spectra with respect to the varying Δ*w* are shown in Figure 6b. Here, Δ*w* is defined as Δ*w* = *w*_1_ − *w*_2_. The new asymmetry parameter can be quantified as *α* = Δ*w*/*w*_1_. As shown in Figure 6b, two new resonances, namely modes III and IV, are exhibited in the transmission spectra with different Δ*w* values. As Δ*w* becomes larger, modes I and II show red shifts in the resonance peaks due to the increase in effective refractive index, while modes III and IV are stable. With the decrease in the difference between *w*_1_ and *w*_2_, the linewidths of modes III and IV become narrower. As *α* approaches zero, modes III and IV become completely invisible in the spectrum and are obviously governed by symmetry-protected BICs. When the symmetry of the x–y plane is broken by the new perturbation, new radiation channels are built for the bound modes and free space; therefore, ultra-high Q-factor resonances governed by quasi-BICs are observed in the transmission spectra. As the asymmetry parameter decreases, the couplings between the bound states supported by the structure and external continuous radiation decrease so that the radiative quasi-BIC modes with ultra-high Q factors are converted into dark BIC modes with infinite Q factors.

The transmission curves for these two modes are also fitted by the typical Fano formula. The obtained Q factors for modes III and IV with different symmetry parameters are shown in Figure 6c,d. The Q factors of modes III and IV also approximately satisfy the inverse quadratic relationship with the asymmetry parameter. As a result, it is possible to obtain ultra-high Q factors by continuously reducing the asymmetry parameter according to the actual demand. Considering the limitations of current fabrication process technology, we calculated that the case of a large asymmetry parameter at Δ*w* is 40 nm, and our proposed structure can still achieve high Q factors of 5.1 × 10^4^ and 1.2 × 10^4^ for modes III and IV, respectively. High Q factors can help to enhance the near-field light–matter interactions in many precision optical devices.

**Figure 7:**
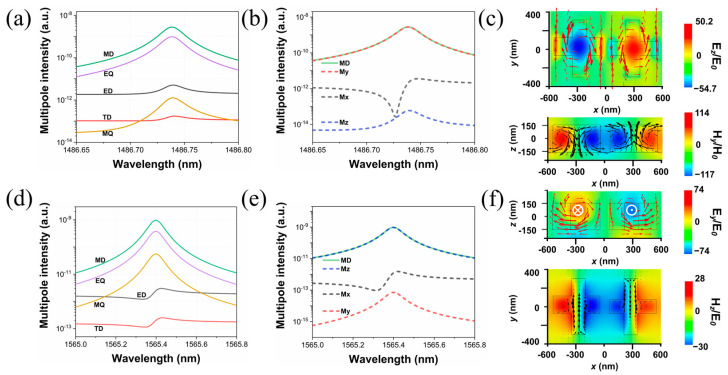
The calculated multipole intensity for modes III (**a**) and IV (**d**) around resonant wavelengths when Δ*w* is 40 nm. The x, y, and z components of MD moments around mode III (**b**) and mode IV (**e**). Electromagnetic field distributions of mode III (**c**) and mode IV (**f**).

We further analyze these two new Fano resonances using the multipole decomposition method in Cartesian coordinates when the Δ*w* is 40 nm. The results are shown as logarithmic coordinates in Figure 7a,d. The dominant multipole components are all MD moments for modes III and IV. The electromagnetic near-field patterns are depicted in Figure 7c,f. The red cones represent the magnetic field vectors, while the black cones represent the displacement current density. As depicted in Figure 7c, in the x–y plane, the magnetic field vectors are mainly distributed in the y direction but also form two closed magnetic vortices in a head-to-tail manner on either side of the central axis, and the displacement current vectors are circulated in a counterclockwise manner between adjacent dielectric crosses in the x–z plane, which satisfies the right-hand screw rule. The components of the MD moment in different directions are calculated in Figure 7b, which also shows that the MD response around mode III mainly originates from the y direction. As for mode IV, the magnetic field vectors form two sets of closed opposite-phase vortices in the x–z plane, and accordingly, the displacement current vectors are circulated in a clockwise manner between the neighboring crosses in the x–y plane. The results of multipole decomposition in different directions in Figure 7e indicate that the MD response of mode IV mainly derives from the z direction.

**Figure 8:**
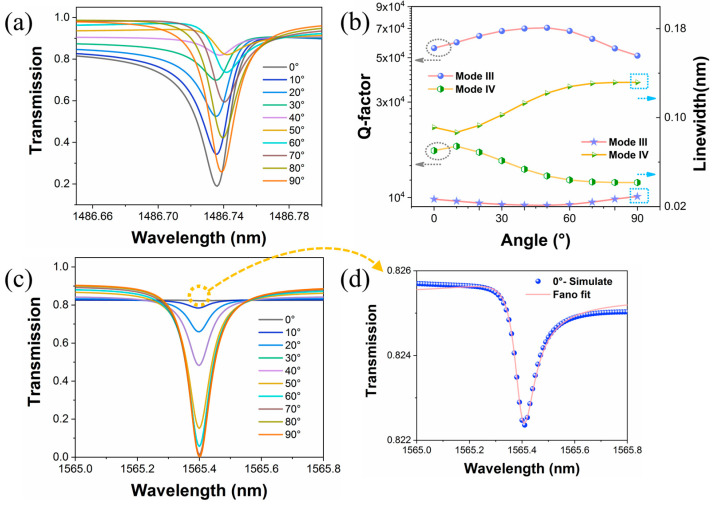
The calculated transmission curves with varying polarization angles from 0° to 90° for mode III (**a**) and mode IV (**c**). (**b**) The Q factors and linewidths for mode III and mode IV at different polarization angles. (**d**) The enlarged transmission curve for mode IV at resonant wavelength and Fano fit curve.

In contrast to the abovementioned metasurfaces that are sensitive to the incident polarization angles [26,31], our proposed metasurface also exhibits robust Q factors towards different incident configurations. Figure 8a,c show the transmission spectra under different polarization angles. The definition of polarization angle is the same as that in Figure 4d. It can be found that the resonance wavelengths in the spectra remain stable at arbitrary incident polarization angles. The Q factors and linewidths of these two modes fitted by the Fano formula also do not fluctuate much within an acceptance range, as shown in Figure 8b. Although mode IV also appears to exhibit similar switching characteristic as modes I and II, thanks to this symmetry breaking, the resonance maintains a high Q factor at 0° polarized incidence, as shown in the enlarged Figure 8d. The stable Q factors are significant for a wide variety of optical devices that depend on light–matter interactions. For example, compared to conventional devices, the freedom from the need for accurate optical calibration can provide more possibilities for relevant applications.

**Figure 9:**
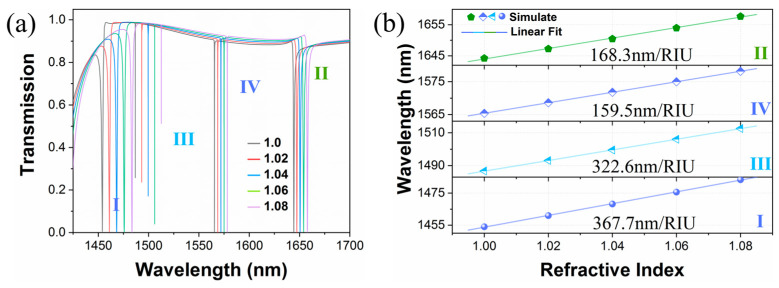
(**a**) The transmission curves with respect to the increasing refractive indices of the surrounding environment from 1.0 to 1.08. (**b**) The calculated resonant wavelengths as a function of medium refractive indices and the linear fit results for four modes.

Finally, we investigate the sensing performance of our proposed metasurface as a refractive index (RI) sensor. As for the previous configuration in Figure 6a, when Δ*L* is 10 nm and Δ*w* is 40 nm, we consider placing the entire metasurface structure in an infinite gas environment with varying refractive indices. The physical domain settings in the simulation are the same as those in Figure 1. For the fabrication technology, the Si film can be deposited on the SiO2 substrate by low-pressure chemical vapor deposition (LPCVD), and the photoresist is spin-coated on the Si thin film and baked. Then, the desired patterns can be obtained by standard electron beam lithography with etching masks and inductively coupled plasma etching. Finally, we can remove the photoresist and clean the sample with deionized water to obtain the proposed metasurface [13]. Figure 9a depicts the transmission spectra with respect to the increasing refractive indices from 1.0 to 1.08. From the sharp Fano resonances, one can observe that the resonant peaks redshift visibly as the refractive index increases, but the linewidths remain stable. In order to evaluate the sensing performance, the sensitivity (S) is a key parameter for the refractive index sensor, which is defined as S = Δ*λ*/Δ*n* (nm/RIU), where Δ*λ* is the shift of the resonance wavelength, and Δ*n* is the change in the refractive index. The sensitivities of the modes are calculated by the slopes of the linear fit curves shown in Figure 9b. Consequently, the values of S for modes I, II, III, and IV are 367.7 nm/RIU, 168.3 nm/RIU, 322.6 nm/RIU, and 159.5 nm/RIU, respectively. As a more comprehensive evaluation criterion, the figure of merit (FOM) is defined as the ratio of sensitivity and the full width at half maximum (FWHM). Given that the linewidths in Figure 9a remain stable, the FWHM is simply calculated from the curve at RI = 1.0. The calculated FOMs of these four modes can reach 234.3, 372.3, 17,922.2, and 1993.8, respectively. We also provide a table of comparison with respect to the sensing performance in Table 2. The sensing performance of our proposed structure is superior to that of other sensors. According to the above analysis, the resonances peak can also be controlled by the surrounding environment so that the applications of the proposed metasurface are more flexible and versatile. In addition, the sensitivities can be further improved by reducing the asymmetry parameter so that higher FOMs can be achieved. Meanwhile, the metasurface with both flexibly controllable multiple Fano resonances and high FOM optical responses is suitable for many multi-parameter sensor devices.

## 4. Conclusions

In summary, an all-dielectric metasurface consisting of two asymmetry crosses in the unit cell is proposed in this work. We achieve the conversion of two resonance modes from BICs to quasi-BICs by introducing in-plane perturbation, where the unit cell of the structure is doubled, while the in-plane symmetry is maintained. Under this dimerization, the Brillouin zone is folded, and the bound modes within it are relocated. We investigate the mechanisms of these two supercavity modes by near-field analysis and electromagnetic multipole decomposition. The flexible tunability and robustness are also analyzed under different conditions. Then, we break the symmetry in both the x and y directions simultaneously to excite two more quasi-BIC modes and achieve multiple Fano resonances. The Q factors of both modes are maintained above 10^4^ with the changes in polarization angles. These modes can be applied to achieve optical switching with a high amplitude modulation range of 95% and refractive index sensing, where the highest FOM can reach up to 17,922.2. We believe that our work can contribute to the study of flexible multimer high-Q devices and enable potential applications in optical switching and multi-parameter sensing, among others.

## Figures and Tables

**Table 1:** Character table for the point C_2*v*_ group.

C_2*v*_	E	C_2_	σ_*x*_	σ_*y*_
A_1_	1	1	1	1
A_2_	1	1	−1	−1
B_1_	1	−1	1	−1
B_2_	1	−1	−1	1

**Table 2:** Comparison of performance.

Reference	Highest Sensitivity	Highest FOM	Application in Gas Sensing	Number of Resonances	Number of Elements
[46]	180 nm/RIU	-	No	1	1
[47]	370 nm/RIU	2846	No	4	2
[48]	295 nm/RIU	738	Yes	4	4
[49]	438 GHz/RIU	3189	Yes	2	4
[11]	379 nm/RIU	1000	No	1	2
[36]	182 nm/RIU	910	Yes	2	4
[50]	-	100	Yes	1	3
This work	367.7 nm/RIU	17,922	Yes	4	2

‘Number of Elements’ represents the number of nanostructures in a periodic unit.

## Data Availability

Data sharing is not applicable to this article.
